# Apomorphia

**Published:** 1901-09

**Authors:** Thomas J. Pugh

**Affiliations:** Bryan, Texas


For Texas Medical Journal.

### Apomorphia.—Its Origin and Therapeutics, as Applied in Asphyxia, Drowning, Lightning Stroke, Emetic in Ejecting Poisons from the Stomach, Labor, Fever, Strangulated Hernia.

BY THOMAS J. PUGH, M. D., BRYAN, TEXAS.

It is the chemist who is generally the pioneer in bringing to the notice of the physician the probable and possible therapeutics of a remedy; but it is often left to the doctor in actual practice, and not unfrequently it falls to the lot of the obscure doctor, hid away in some out-of-the-way section, to bring forth the new or the old remedy to a fuller realization of its value.

In searching our literature on the subject of apomorphia we find in our U. S. D. (16th edition, page 215, and 17th edition, page 186) that apomorphia was discovered by Dr. Mathieson and Mr. Wright.

This preparation is made by heating morphine in a closed tube, with a great excess of hydrochloric acid, for two or three hours, at a temperature of 140° to 150°.

The contents are then treated with bicarbonate soda and the precipitate exhausted with ether or chloroform.

Apomorphia may also be made by the action of hydrochloric acid on codiene, and it is affirmed that the best method in practice is that of E. Meyer, in which morphine is treated with a solution of zinc chloride at 120°.

Just here I would suggest that oftentimes the doctor wants a quick and unfailing emetic, and to this end, it might be well to have our chemist to add to his apomorphia, emetine, which you all know, in suitable doses, to be a reliable emetic.

With this prelude, I propose to go at once into the therapeutics of apomorphia.

---

*Read before the Brazos Valley Medical Association, at its eleventh semi-annual meeting, at Calvert, Texas, May 15, 1901.

Its sphere of usefulness is indeed far reaching, and I am persuaded that if used opportunely, many valuable lives might be saved which otherwise "perish from lack of knowledge" of the value of this simple remedial agent.

In asphyxia, from any cause, where life seems to be extinct, and where we must have respiration to put the wheels of life in motion, apomorphia, in one-tenth grains every ten minutes, given hypodermically, in the arm or over the stomach, is the sheet anchor of the physician. In such emergencies, of course if death is so profound that reflex action is destroyed, neither apomorphia nor any other remedial agent will avail anything.

In 1893, it was my pleasure to meet with the N. A. Railway Surgeons at Omaha. During my stay there, one morning at 8 o'clock, Drs. Tally, Dewey and myself were summoned to an upper story of the Barker Hotel, to see a man who had come in the night before from the west, and on retiring to his room at eleven o'clock had failed to turn out his gas, but had blown it out and gone to sleep. Next morning the doctors, myself among the number, found him profoundly asphyxiated from the escaping gas. The two physicians with me, after examining the man, said he was dead beyond all hope, and as we started to retire from the room, I asked one of the doctors if he had a hypodermic syringe and a vial of apomorphia, to which he responded in the affirmative. The syringe was handed to me, and I at once gave the patient one-tenth grain of apomorphia, hypodermically. Within two minutes, the man was vomiting freely, the diaphragm was acting, respiration was re-established, the heart's action was restored, and the man who a few moments before was more dead than alive was on the road to a safe recovery, in thirty minutes being out of danger, and two hours after he left the hotel, completely restored.

In drowning, I would commend this drug, hypodermically, as it restores suspended respiration, and does away with the necessity of artificial respiration.

I believe this would be the appropriate remedy in lightning stroke. I also think blood-letting should be resorted to in addition to the use of this remedy. I am persuaded that in the lightning stroke we have a more complete stasis of the blood current, and therefore I recommend blood letting as an aid in reestablishing the circulation of the blood, and it must be evident to all that blood letting will, to a certain extent, relieve the overgorged organs and tissues, thereby aiding pulsations and respiration.

In morphine of opium poisoning, in any of its forms, apomorphia, hypodermically, is the remedy. I visited a woman in my town who, with suicidal attempt, had, some ten minutes before I saw her, swallowed twenty grains of morphine. She was as black as a black hat; she was deeply narcotized. I had to resort to my remedy, apomorphia, and in five minutes vomiting set in, her respiration and circulation grew better, and the woman was saved.

It is the remedy, at least one of the valuable remedies, in retarded labor, due to rigid os. The obstetrician will value it when he has used it. It is useful in stubborn fevers, sometimes aborting them as by magic.

Now, last but not least, we come to its use in strangulated hernia. We have a case before us; we have used every effort in finger manipulation; we have even given chloroform and tried to reduce the constricted gut. We have failed. What next? The knife? No; let us give the patient one-tenth grain apomorphia hypodermically. We get vomiting in five or ten minutes; if not, repeat the dose. The patient vomits freely. Place him head downward, raise his hips, and if he is not inclined to sleep give him one-fourth of a grain of morphine, hypodermically. The patient goes to sleep and rests perhaps for an hour; when he awakes, the gut is in its proper place. Relaxation due to the vomiting, sleep induced by the morphine, together with gravitation, does the work.

I have treated quite a number of strangulated cases in this way, and always successfully. I have never yet failed in the accomplishment of the ends desired with this drug, in strangulated hernia.

